# Patch test of dental materials in Oral Lichen Planus with considering the role of saliva

**DOI:** 10.1038/s41598-021-87778-8

**Published:** 2021-04-15

**Authors:** Farzaneh Agha-Hosseini, Elahe Gholamrezayi, Mahdieh-Sadat Moosavi

**Affiliations:** 1 Dental Research Center, Dentistry Research Institute, Tehran University of Medical Sciences, Tehran, Iran; 2 Department of Oral Medicine, School of Dentistry, Tehran University of Medical Sciences, Tehran, Iran; 3 The Academy of Medical Sciences, Tehran, Iran; 4 Department of Orthodontics, School of Dentistry, Tehran University of Medical Sciences, Tehran, Iran

**Keywords:** Immunological disorders, Oral diseases

## Abstract

Lichen planus is the most common skin disease that affects the oral mucosa. Oral Lichen Planus is a T-cell-mediated autoimmune disorder. In the current study, for the first time, an oral cavity condition in skin patch tests with adding saliva is simulated. In addition, the patch results are compared with healthy subjects. Forty-one OLP patients and 63 healthy subjects were enrolled in the study. All participants were provided with patch tests, including allergens, in combination with saliva in chambers. Allergens from the European baseline (standard) series selected according to the most prevalent positive results in the previous study were applied. Positive results of Mercury and Cobalt tests were significantly higher in the case group. In this study, the differentiation of patients with lichen planus and lichenoid was identified according to the Van der Meij & Van der Waal criteria. The patch test was conducted for healthy individuals as well. The most important of all was the use of patients' saliva in the patch test, done for the first time in this field. In the case of OLP, a patch test can help identify positive reactions to dental materials; thus, the replacement of dental restorations may be needed.

## Introduction

Lichen planus is a chronic mucocutaneous disease mediated by the immune system. It often affects oral mucosa and has a prevalence of 1–2%. About 15% of patients with Oral Lichen Planus (OLP) also have skin involvement. In other words, lichen planus is the most common skin disease that affects the oral mucosa. OLP is found more frequently among women, and it is diagnosed between the age of 30–60 more than any other age^1^.

It has various oral manifestations, including reticular, popular, plaque-like, erosive, and atrophic types^2^. The reticular form is the most common type^3^.

The exact etiology of OLP is still unknown, although it is a T-cell-mediated autoimmune disorder in which CD8 + T-cells induce apoptosis of the oral epithelial cells^4^. Some Etiological factors have been proposed for this situation, including bacterial, fungal, and viral infections (e.g., hepatitis C), local trauma, psychological stress, galvanic phenomena, immunological defects, and allergic reaction to dental materials, such as dental alloys, amalgam, and mercury^5,6^.

As there is no definitive cure for OLP, the first goal of treatment is to subside the symptoms of the disease. To date, Systemic and topical corticosteroid therapy has been the most effective approach to reach this goal; however, the side effects of these kinds of medications have limited their use. Indeed, some patients are refractory to this treatment and cannot tolerate the side effects^4^.

Several studies have reported the OLP possibility for malignant transformation. Its malignancy transformation risk has been reported between 0 and 3.5%, among which erosive and atrophic types have the highest risk^1^. The World Health Organization (WHO) categorizes OLP as potential malignant lesions and recommends patients with it to be closely monitored^7^.

Cutaneous irritants are the main reason for contact allergic reactions. Therefore, the identification of these stimulants is of great importance in the treatment of these lesions. One manner to identify these stimulants is animal studies; however, there are many differences in animal responses to stimuli compared to human beings^8^.

The patch test, introduced as a diagnostic method in the late nineteenth century, is widely used as a simple non-invasive method for detecting contact allergic reactions^9^. Patch tests are used as an auxiliary diagnostic method in many oral diseases, including lichen planus, lichenoid lesions, oral burning syndrome, and mucous membrane pemphigoid^10^.

Clinical manifestations of contact allergic reaction to dental materials differ from tenderness or burning sensation to lesions like stomatitis and lichenoid reaction. Patch testing may help identify these types of hypersensitivity to commonly used dental materials^11^.

As mentioned above, oral mucosa has intimate contact with allergens and stimulants. Amalgam, composite, other resin-based materials, and metals, such as nickel, are some of these stimulants responsible in Oral Lichen Planus pathogenesis^12,13^; their removal and replacement may have an impressive outcome in affected patients^14^.

Several studies have tried to figure out the clinical importance of oral diseases and contact allergy to dental materials. Some contradictory results have been seen in some of the studies^15,16^; however, the reliability of patch testing in the diagnosis of oral diseases, especially Oral Lichen Planus, has been confirmed in some^12^. In the current study, for the first time, an oral cavity condition in skin patch tests with adding saliva is simulated. In addition, the patch results are compared with healthy subjects.

## Methods

The present study was performed under the approval of the Tehran University of Medical Sciences Ethics Committee, which is following the Declaration of Helsinki (DENTISTRY.REC.1396.3048). Written informed consent was obtained from all participants. The research was done in a case-control manner. Due to the lack of the study with the same methods, a pilot study was implemented to determine the minimum sample size needed to obtain reliable and valid results. Among referral patients with erosive-atrophic OLP to the department of oral maxillofacial medicine, a group of 41 patients with the inclusion criteria was chosen for our experimental group.

### Subjects

Inclusion criteria, including OLP diagnostic confirmation, were evaluated based on Van der Meij & Van der Waal criteria^17^. This criterion differentiates between lichenoid and lichen planus lesions, and only lichen planus patients were included in this study; any other lichenoid-associated lesions were considered as exclusion criteria. Exclusion criteria involved: (1) signs of dysplasia in histopathology, (2) lichenoid drug reaction, (3) pregnancy, (4) breastfeeding, (5) atopic dermatitis, (6) seasonal allergy, (7) intake of immunomodulator immunosuppressant, (8) intake of desensitizer drugs, and (9) the presence of similar lesions adjacent to amalgam restorations. A control group of 63 healthy subjects, who lacked exclusion factors, was chosen among university personnel and companion patients. Each one was provided with a patch test, including ten chambers (for applying allergens) from Chemotechnique diagnostic company. Since different amalgam brands have different compositions and proportions of metals, the metals in amalgam and dental alloys were used separately in patch testing. Allergens from the European baseline (standard) series selected according to the most prevalent positive results in the previous study^11^ were applied as follows:

- Mercury 0.5%
- Cobalt Chloride Hexahydrate 0.1%
- Nickel Sulfate Hexahydrate 0.5%
- Palladium Chloride 2%
- Sodium Tetrachloropalladate Hydrate 3%
- Potassium Dichromate 0.5%
- Menthol 2%
- Benzoyl Peroxide 1%
- Hair dye

The chamber for mercury was separated from other ones. Patients were asked not to eat and drink 90 min before saliva collection. Non-stimulated saliva was collected in plastic vials by spitting. Saliva was then centrifuged (2500*g*, 10 min), and supernatants of saliva were separated. The patients' saliva was poured into all holes.

The mentioned allergens were applied to holes of the patch test. Then, it was placed on the shoulder of patients. The results of the mercury patch test were evaluated after 72 h, and those of other allergens were evaluated after 48 h by an oral maxillofacial medicine specialist trained in patch test reading and using a patch test reader.

The guide for results evaluation:

- Negative reaction (−): non-revealing symptoms
- Weak positive reaction (+): erythema, papules, infiltration
- Strong positive reaction (++): erythema, papules, infiltration, discrete vesicles
- Extreme positive reaction (+++): coalescing vesicles, bullous or ulcerative reaction

### Statistical analysis

For data analysis, SPSS software (Chicago, IBM) version 25 was used. To draw a comparison on sensitivity to different allergens between the experimental group and the control group, the Chi-square test (and Fisher test if needed) was used.

## Results

**Figure 1:**
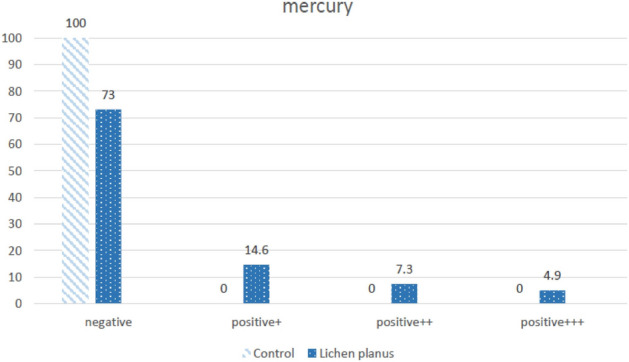
Results of mercury tests in Oral Lichen Planus and control groups.

**Figure 2:**
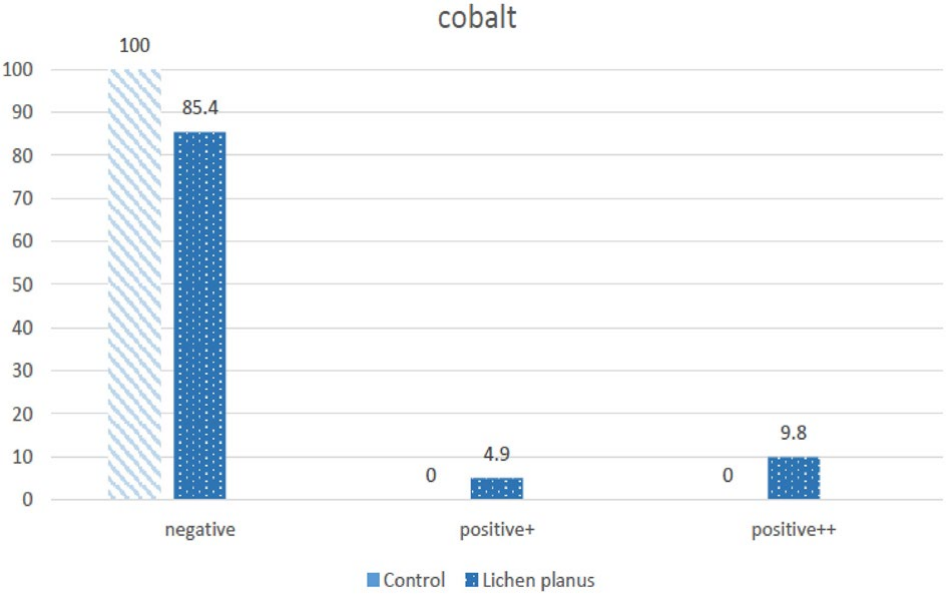
Results of cobalt tests in Oral Lichen Planus and control groups.

In this study, a case group of 41 OLP-affected patients and a control group of 63 healthy subjects and dentistry personnel were selected. In the OLP group, the male to female ratio was 28 to 13 and in the control group 49 to 14. The mean age in the OLP group was 53.76 ± 11.89 (Min: 28, Max:76) and in the control was 46.02 ± 12.68 (Min: 49, Max:14). The comparison of patch test results is summarized in Table 1. None of the healthy subjects were positive in Mercury, Cobalt, Nickel, Potassium, Menthol, and Benzoyl Peroxide. Positive results of Mercury and Cobalt tests were significantly higher in the case group (Figs. 1, 2). Based on the potential impact on lesion healing, patients with positive results were referred for material replacement.

**Table 1:** Patch test results.

Allergens	Case (n = 41) (%)	Control (n = 63) (%)	*P* value
Mercury 0.5%	26.8	0	< 0.001
Cobalt Chloride Hexahydrate 0.1%	14.6	0	0.003
Nickel Sulfate Hexahydrate 0.5%	4.9	0	0.15
Hair dye	14.6	4.8	0.55
Palladium Chloride 2%	29.3	28.6	0.94
Sodium Tetrachloropalladate Hydrate 3%	43.9	34.9	0.11
Potassium Dichromate 0.5%	4.9	0	0.15
Menthol 2%	4.9	0	0.15
Benzoyl Peroxide 1%	2.4	0	0.39

Analysis were done with Chi-square test (or Fisher exact test if appropriate).

The negative response to the mercury patch test was seen in 63 members of the control group, while in 41 patients with Oral Lichen Planus, an 8.62% positive reaction was observed. Among 63 members of the control group, 56 of them had amalgam filling; none of them showed hypersensitivity to amalgam mercury. This result was achieved while 31 members from 41 patients with Oral Lichen Planus have an amalgam filling, and the mercury test was 8.26% positive in 22 of them.

## Discussion

Oral mucosa is exposed to different stimulants and allergens constantly, although contact reaction lesions occur less often in the oral mucosa. The reason can be partial resistance of oral mucosa due to anatomic, physiologic, and immunologic conditions. The abundance of oral mucosa's blood vessels and dilution of allergens by saliva prevent the long-term contact of stimulants with mucosa and reduce allergens' absorption. Moreover, a low number of Langerhans cells and T-lymphocytes decrease this reaction. The reaction due to contact with stimulants in oral mucosa occurs via immunologic procedures led by T-helper lymphocytes. The physiopathology of this reaction is similar to contact dermatitis and should be considered an allergic contact reaction.

Some diseases are related to contact sensitivity, such as allergic contact stomatitis, allergic contact cheilitis, geographic tongue, OLP, and secondary burning mouth syndrome^18^. Considering T-helper1 lymphocytes' role in the etiology of OLP, allergic contact reactions may be involved in the pathogenesis of OLP; however, the relation between OLP and contact sensitivity has not been confirmed in any studies.

In contrast to lichen planus skin lesions, which are usually self-limited, oral lesions are chronic, and self-regression rarely occurs. OLP typically affects middle-aged individuals. It mostly affects women^19^, parallel to studies carried out in the field of oral allergies^20^.

In previous studies, the role of dendritic cells in the presence of different antigens causing immunologic reactions has been mentioned. In addition, some studies have suggested the implication of allergens (e.g., materials used in dentistry) in stimulation (Activation) of APCs^5^.

Oral Lichen Planus has a great resemblance to Oral Lichenoid Reaction; actually, they are histologically and clinically similar, and the differential feature is the etiology of lesion development. The patch test can be used not only as an adjunctive tool to distinguish Oral Lichenoid Reaction from Oral Lichen Planus but also as a treatment^21^.

Patch testing is diagnostically valuable in patients who suffer from allergic contact dermatitis and other types of mucocutaneous delayed hypersensitivity. It is useful in ruling out contact allergy from possible differential diagnosis of various disorders and demonstrating the body reaction in response to contact allergens^22^.

Several investigators have used the patch test to identify allergens in dental materials^11,15^. Kim et al. have stated the worth fullness of the patch test in oral disease evaluation, especially for Oral Lichen Planus^12^.

In the current study, the patch test was done for OLP patients based on the following approach that may explain the differences with previous studies:

1. According to Van der Meij & Van der Waal criteria, Oral Lichen Planus and Oral Lichenoid Reaction have been distinguished^17^.
2. Patch test results of patients were compared with healthy individuals.
3. As manifestations of delayed hypersensitivity to mercury usually appear after 72 h^23^, the result of Mercury has been assessed after 72 h.
4. Patients' saliva is included in allergens chambers in this study to simulate the oral cavity conditions.

The oral cavity contains a biologic substance, saliva, in which minerals, electrolytes, proteins, and enzymes are dissolved. The number of proteins and glycoproteins in the whole saliva, mostly at minute concentrations, is up to 3000^24^. Saliva and its components can play a role in antigens and haptens conversion, also in the release of stimulants and allergens from dental materials in the oral cavity. Thus, there is an interaction between dental materials and physiologic fluids of the oral cavity. Oral tissues are exposed to a real bombardment of both chemical and physical stimuli, as well as the metabolism of many bacterial species; however, oral tissues remain healthy most of the time. Dental restorations, including dental implants, should endure in this interactive environment. Dental materials in the oral cavity may undergo electrochemical corrosion due to their temperature and PH fluctuations. Allergic reactions may be due to the presence of ions produced by the corrosion of restorations and implants^25^.

According to Koppelman et al., the interaction between allergens and the immune system may be influenced by saliva^26^. Therefore, patients' saliva is included in allergens chambers as a major difference between skin and oral mucosa is the presence of saliva in the oral cavity^27^. One other ability of saliva in modifying the biologic process is the differences in skin wound healing compared to the oral cavity, with faster, more regenerative mechanisms along with reduced inflammation. Saliva is known to be a critical part of oral homeostasis^28^. Therefore, if the skin patch tests are carried out without the presence of this important biological substance, the interaction of proteins and mediators of saliva on immune responses is ignored.

Keeping the saliva in contact with the skin is safe. Firstly, every person's saliva is used for his or her healthy skin. Second, saliva is an ultra-filtrated fluid from serum and contains anti‐microbial properties and peptides with complementary functions^27^.

Based on the methodological differences, in the current study, 70% of Oral Lichen Planus patients reacted positively to at least one of the dental materials-related allergens; in contrast, in the previous study, 38% of 21 assessed patients (including 16 treatment-resistant Oral Lichen Planus and 5 atypical lichenoid lesions) demonstrate positive response. Moreover, 27% of 115 patients (including 94 Oral Lichenoid Reaction, 16 treatment-resistant Oral Lichen Planus, and 5 atypical lichenoid lesions) react positively to amalgam mercury^11^. Mobacken et al. stated 16% positive reactions to the mercury component in Oral Lichen Planus patients^29^; this contrast could be attributed to longer exposure to mercury-containing patch test. Therefore, it seems rational to evaluate the results of patch testing at least 72 h from its initial contact.

In 2003, Thornhill et al. examined the relationship between amalgam restorations and OLP. The results of the patch test for amalgam or mercury were positive in 3.9% of OLP patients^30^. Perhaps the difference between the present research and Thornhill's study can be explained based on the OLP diagnostic criteria. In this study, the OLP diagnosis was based on Van der Meij & Van der Waal Criteria while in that of Thornhill on the Eisen criteria.

Several studies have suggested that replacing amalgam in OLP patients can reduce signs and symptoms. Especially if the mercury patch test results, as the most important allergen of the amalgam, are positive^31^.

Regarding these studies and the results of the mercury patch test in the present study, it is anticipated that by replacing amalgam restorations in patients with the positive patch test result recovery from OLP is likely to be achieved.

In the current work, in 63 cases from the control group, the test result for cobalt was negative. However, the results of the reaction test to cobalt in the lichen planus group of 41 cases were positive in 6 cases (14.6%), showing a significant difference between the control and experimental group.

Similarly, in a study by Hammouda et al. in 2015, the results of the cobalt patch test in a normal healthy population were 2.96%, while in the OLP group, it was 32.2%, significantly higher than the normal population^10^.

In a study, Mobacken performed a patch test for 67 OLP patients and 50 normal individuals. The result of the cobalt patch test was 2.9% in the OLP group and 14% in the control group, of which the difference was statistically significant^29^. The reason for this difference could be the standard used to diagnose OLP. The Mobacken study did not select patients based on Van der Meij & Van der Waal Criteria. Of course, the most important reason for justifying a higher percentage of positive results of the cobalt patch test in the present research is the application of patients' saliva in the patch test.

According to the present study, it seems that the existing mercury in amalgam can play a role in the onset of this disease, although removal of this substance does not lead to a 100% recovery in OLP patients. In this study, since in addition to mercury, cobalt in alloys used in restorations, have caused positive patch test result, may have an important role in delayed healing in patients with Oral Lichen Planus after the removal of amalgam restorations.

As mentioned earlier, in this study, the results of the cobalt patch test in OLP patients are significantly higher than the control group. This is even though most previous studies have focused on amalgam and its mercury compounds, while cobalt is used in various prosthetics, such as fixed and removable, as well as implants. It is recommended to use a patch test for those patients with Oral Lichen Planus that have amalgam restorations before replacing amalgam restorations with metal-ceramic or new restorations; thus, in the case of sensitivity to cobalt, alternative alloys can be used instead.

Based on the present study and the trace of the mercury and cobalt in the pathogenesis of OLP, a patch test is suggested to be performed in patients without autoimmune disease before using mercury and cobalt-containing substances in order not to expose vulnerable patients to OLP. Also, possibly the replacement of restorations and prostheses containing these two elements with other substances may be effective in the recovery of OLP patients whose patch test results are positive for these two elements.

One of the limitations of this study was the lack of a control group in which allergens could be placed in chambers without saliva. Since previous studies have tested the same allergens in saliva-free chambers, the study results were compared with them.

In the past, the role of hypersensitivity reaction was known in the OLR pathogenesis. In this study, in which precise differentiation between OLP and OLR was considered, the role of hypersensitivity reaction in OLP disease was more pronounced. This illustrates a more prominent role of the patch test in the management of OLP patients.

Therefore, in the case of OLP, the patch test can be helpful as the first step in identifying positive reactions to dental materials. Then, the replacement of dental restorations should be performed considering patients' consent, and the results should be evaluated after a while.

It is suggested that patch testing can change the treatment priorities, thereby reducing the use of medications, each of which in turn has side effects. Similarly, the patch test can prevent OLP from becoming a chronic disease, thereby reducing the risk of malignant transformation of OLP.

## Conclusion

In general, the present study has advantages over previous ones. In this work, patients with lichen planus and lichenoid are differentiated according to Van der Meij & Van der Waal Criteria. This is very important since these two diseases have a different nature. The patch test is conducted for healthy individuals as well, and its results are compared with the results of patients with lichen planus. In addition, exposure to mercury allergens is performed for 72 h. The most important of all is patients' saliva use in the patch test, done for the first time in this field.
